# Influence of Artificial Turf Surface Stiffness on Athlete Performance

**DOI:** 10.3390/life10120340

**Published:** 2020-12-10

**Authors:** John Wannop, Shaylyn Kowalchuk, Michael Esposito, Darren Stefanyshyn

**Affiliations:** Human Performance Lab, Faculty of Kinesiology, University of Calgary, 2500 University Dr NW, Calgary, AB T2N 1N4, Canada; shaylyn.kowalchuk@ucalgary.ca (S.K.); michael.esposito@ucalgary.ca (M.E.); darren.stefanyshyn@ucalgary.ca (D.S.)

**Keywords:** artificial turf, performance, sport biomechanics

## Abstract

Properties of conventional playing surfaces have been investigated for many years and the stiffness of the surface has potential to influence athletic performance. However, despite the proliferation of different infilled artificial turfs with varying properties, the effect of surface stiffness of these types of surfaces on athlete performance remains unknown. Therefore, the purpose of this project was to determine the influence of surface stiffness of artificial turf systems on athlete performance. Seventeen male athletes performed four movements (running, 5-10-5 agility, vertical jumping and sprinting) on five surfaces of varying stiffness: Softest (−50%), Softer (−34%), Soft (−16%), Control, Stiff (+17%). Performance metrics (running economy, jump height, sprint/agility time) and kinematic data were recorded during each movement and participants performed a subjective evaluation of the surface. When compared to the Control surface, performance was significantly improved during running (Softer, Soft), the agility drill (Softest) and vertical jumping (Soft). Subjectively, participants could not discern between any of the softer surfaces in terms of surface cushioning, however, the stiffer surface was rated as harder and less comfortable. Overall, changes in surface stiffness altered athletic performance and, to a lesser extent, subjective assessments of performance, with changes in performance being surface and movement specific.

## 1. Introduction

Artificial turf surfaces have risen in popularity in recent years, with estimates of over 6000 artificial surfaces installed in North America, and 1000 to 1500 new installations each year [1]. Artificial turf systems are composed of individual components that can be independently manipulated to alter the mechanical properties of the playing surface to desired levels. These components generally include an underlayment or shock pad as well as the surface layer that the athlete is in contact with composed of (1) a carpet with synthetic fibers and (2) infill material consisting of combinations of sand, rubber and possibly other organic materials. 

Manipulating stiffness of athletic surfaces has shown potential in terms of increasing athletic performance [2,3,4,5] and reducing injury risk [6]. During sport, athletes will deform the surface by exerting large forces during sprinting, running and cutting, with peak contact forces reaching over 2000 N [7]. As the surface deforms, it will store potential energy that is transferred from the athlete, returning this energy to the athlete when they leave the surface. Ideally, all the energy stored in the surface will be returned to the athlete, however, realistically, some energy will be lost as heat, sound and vibration which will not benefit the athlete.

Energy loss is influenced by the properties and components of the sport surface. Mechanical drop tests on conventional running surfaces have shown energy loss ranging from 50% to 78% depending on the specific surface [8]. Due to the rubber and sand materials used in conventional infilled artificial turf surfaces designed to cushion landings rather than optimized for the storage and return of energy, energy loss upwards of 85% has been quantified on these surfaces [8]. However, recent advancements in infill material and underlying shock pads of artificial turf systems allow for the optimization of the surface stiffness. Even subtle changes to the amount of energy loss on artificial turf systems may result in noticeable improvements in human performance. For example, if the amount of energy loss of artificial turf systems can be reduced to 75%, this may be substantial enough to influence athletic performance. The total mechanical energy required for a running stride is between 100–200 J [9,10], and if the surface can store 12 J of energy during a sprinting stride [11] energy return of 3 J would represent over 1.5 percent of the mechanical energy per stride and could potentially result in a quantifiable increase in performance. Improvements in performance as described above have been accomplished on specially designed running surfaces, with optimal surface stiffness being determined and performance increases between 1–3% being predicted through models [2,3]. These predictions were then verified, as a running track built with properties based on these model predictions resulted in running performance enhancements of 2%.

Aside from direct storage and return of energy by the surface, the stiffness of the surface can also have a direct influence on the movement patterns and energy expenditure of the athlete. Athletes will alter their biomechanics when interacting with different surfaces [9,10]. Internally, muscle tendon characteristics and the force generation of muscles is also altered with changes in the stiffness of the surface [4,12]. These changes in athlete movement patterns and energy expenditure have resulted in direct changes in athletic performance, with both absolute jumping performance [12] and running economy being altered with changing surface stiffness, and magnitude of performance increases as high as 12% being reported [5].

It appears that both short duration (sprinting, vertical jumping) and long duration (running) performance can be influenced by changes in surface stiffness. While these results are positive, it should be noted that many of these studies utilized extreme changes of stiffness or had athletes performing very controlled non-sport specific movements. When considering the stiffness of artificial turf systems, studies have shown that varying stiffness can influence peak vertical accelerations during running [13] as well as athlete contact times and step lengths [14]. Thus, it seems reasonable to assume that variations in infilled artificial turf stiffness could directly influence athletic performance, but this has yet to be determined. Furthermore, the underlying mechanism of how changes in surface stiffness affect athlete performance and biomechanics remains unknown making it difficult for sport surface manufacturers to optimize surface stiffness.

Therefore, the purpose of this project was: (i) to determine the influence of surface stiffness of artificial turf surfaces on athlete performance; (ii) to investigate changes in kinematics on surfaces that have altered performance to gain insight into the mechanisms involved in any performance changes.

## 2. Materials and Methods

### 2.1. Participants

Seventeen male competitive football and soccer athletes were recruited for the experiment (81.6 ± 11.1 kg, 181.9 ± 8.69 cm 26.4 ± 3.0 years). Informed written consent was obtained from all participants prior to data collection, with the study being conducted in accordance with the Declaration of Helsinki, and the protocol approved by the University of Calgary’s Ethics Board (REB18-0941). All participants were provided with an adidas X19.3 cleat (Herzogenaurach, DE) in either a US men’s size 9, 10, 11 or 12.

### 2.2. Surfaces

The five different artificial turf surface conditions that were used in the investigation were constructed and installed outdoors over an asphalt surface, with the stiffness of the surfaces altered through changes in shock pad material (surfaces 1–3) or changes in carpet and infill material (surfaces 4–5).

All surfaces contained the FieldTurf (FieldTurf, Tarkett Inc., Montreal, QC, Canada) Classic HD 2.5” base carpet with carpet for surfaces #1–4 having a fiber density of 1220 g/m^2^. Surfaces #1–4 had identical carpet and infill compositions, with the only differences between them being their shock pad material (or lack of shock pad for surface 4). Surface #5 did not contain an underlying shock pad, with changes in stiffness being attained through differences in both the infill composition and density of the carpet fibers (lower density of fibers −1220 g/m^2^). Surface 4 was termed as Control, due to it being the common surface installed by the manufacturer for high performance sporting activities.

Following construction of all turf surfaces, mechanical drop test measurements were performed following modified American Society for Testing and Materials (ASTM) standards F355 [15] and F1936 [16]. Modifications to the testing protocol consisted mainly of a reduction in the number of drops on the smaller sections of artificial turf. Five drops were performed for each surface with the mean values of the peak deceleration quantifying the stiffness of each surface. Through alterations of the shock pad and modifications of the carpet/infill material systematic changes in stiffness were achieved. The specifics of the surface constructions are shown in Table 1.

### 2.3. Data Collection

Data collection consisted of two testing sessions; during session one running economy was measured, while during session two sprint, agility and jump performance was measured on the different surfaces.

**Figure 1:**
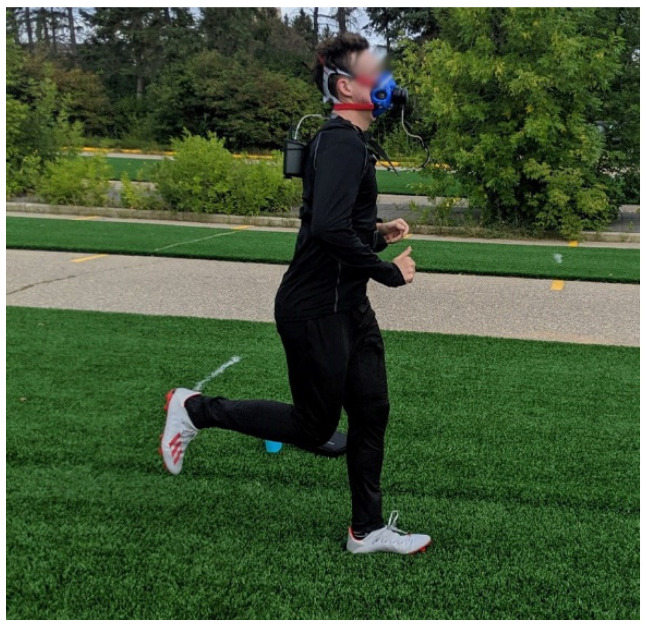
Photograph of test setup of the Cosmed on the artificial turf surfaces.

During the running economy session, athletes were outfitted with a Cosmed K5 breath-by-breath metabolic analysis system (Cosmed, Rome, Italy). As data was collected outdoors, a recent study has shown the Cosmed K5 to be valid for measuring submaximal gas-exchange variables in an outdoor environment [17]. Immediately before each running economy session, the Cosmed O_2_ and CO_2_ sensors were calibrated with a gas containing known concentrations of O_2_ and CO_2_ following the Matheson Certified Calibration Standard. The final calibration step involved calibrating the sampling turbine with a 3.0 L syringe (Hans Rudolph Inc., Kansas City, MO, USA) to ensure accurate volumetric measurements. When the participant was ready to begin their warmup, they were outfitted with a heart rate monitor and a breathing mask (Hans Rudolph Inc., Kansas City, MO, USA) connected to the Cosmed (Figure 1). The Cosmed calculated the volumes of O_2_ and CO_2_ consumed and produced at every breath and averaged these values every 10 s.

The test session began with an incremental running test to a submaximal intensity. The participant began running at 2.5 m/s and increased their speed by 0.22 m/s every 2 min. Running speed was controlled by a computer program that output loud audible beeps set to coincide with when the participant should be reaching the end markers (20 m apart) labelled on each turf surface. The participant continued running at faster speeds until they reached anaerobic threshold (AnT). The identification of AnT uses multiple variables to provide an understanding of exercise intensity but the most commonly used variables during testing were (1) a non-linear increase in pulmonary ventilation and (2) the point of excessive CO_2_ production [18]. Once CO_2_ production consistently exceeded O_2_ consumption, this indicated that the exercise intensity associated with AnT had been reached. This incremental test served two purposes: (1) it identified the speed at which each participant would run during data collection, which was identified as 1.0 mph below the speed at which AnT was reached and (2) it allowed the participants to warm-up prior to data collection.

After completing this incremental test, the participant was allowed to rest for ten minutes. Then a series of five five-minute constant speed running trials were performed, one on each surface, with five minutes of rest between each trial. The order of surfaces that the trials were performed on was randomized. For each running trial, the average O_2_ consumption over the last two minutes of the trial was calculated for analysis.

During the second testing session, participants performed three performance drills on each of the different surfaces: 5-10-5 agility drill, 10 m sprint acceleration and running vertical jump.

**Figure 2:**
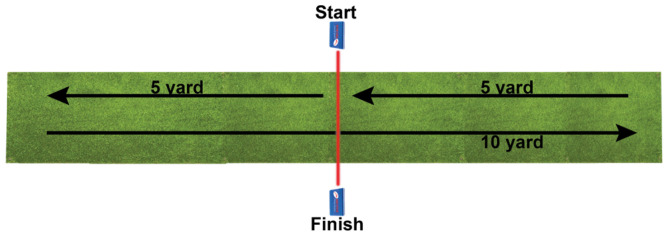
Schematic of the 5-10-5 agility drill.

The 5-10-5 agility drill had the athlete starting by straddling a photocell timing light. When instructed to begin, the athletes turned 90° and burst forward 5 yards, then turned 180° and moved in the opposite direction for 10 yards before again turning 180° and reversing direction and sprinting 5 yards back to the starting position (Figure 2). Brower timing lights were placed in the center of the drill and measured the start and end time. Agility drill performance was defined as the time to complete the drill.

**Figure 3:**
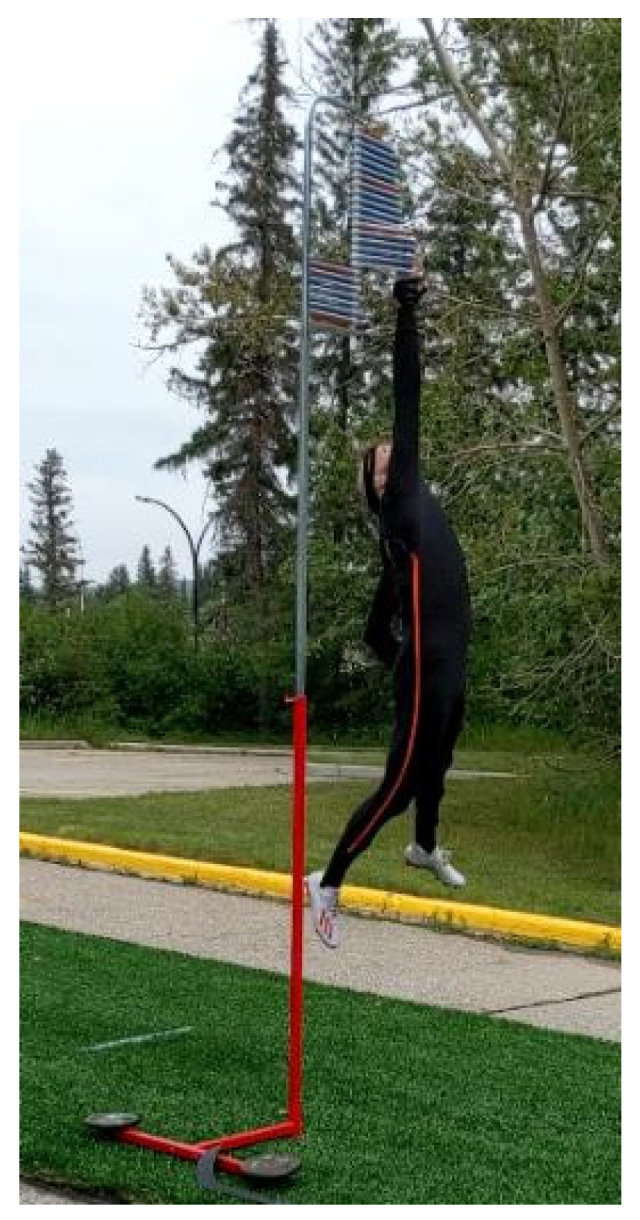
Photograph of a participant performing the maximum jump test.

The maximal effort vertical jump was performed with a Vertec jump meter, with the athletes being instructed to jump as high as possible. Participants were allowed to perform any approach they desired with the goal of reaching as high a level as possible on the jump meter. Participants were provided with enough practice trials in order to attain their preferred jumping technique and were required to maintain this technique throughout the duration of the testing session (Figure 3).

**Figure 4:**
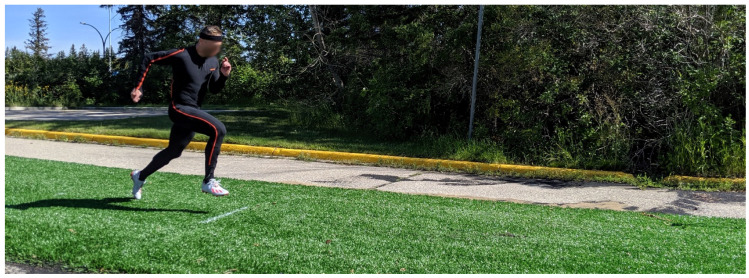
Photograph of a participant performing the 10 m sprint acceleration.

Lastly, participants performed a maximum effort 10 m sprint acceleration. Athletes started in a stationary, self-selected stance at the starting line, accelerated forward while breaking the starting line timing gates and sprinted 10 m through the finish line timing gates (Figure 4). Sprint acceleration performance was determined as the time to sprint 10 m.

Three trials were collected during each drill on each of the five surfaces, with the athletes also performing practice trials of each movement prior to testing to mitigate learning effects. As with the running session, the surfaces were used in a randomized order and the mean of the three trials on each surface was used for comparisons.

Biomechanical data were also recorded at 240 Hz during the second testing session (sprint/agility/jump) using a full body inertial motion capture system (Xsens, Xsens Technologies BV, Enschede, The Netherlands). The motion capture system consisted of 17 motion trackers placed at anatomical locations on the body with each tracker containing a 3D linear accelerometer, 3D rate gyroscopes, 3D magnetometers and a barometer. Following placement of the trackers, a neutral pose of the athlete was taken that defined the anatomical axis while also determining the relative orientation/location of each tracker. Following completion of the calibration pose, a joint coordinate system was established allowing for 3D kinematics to be measured during each movement. Following data collection, joint angles and foot contact phases were calculated within MVN Analyze 2020.0.2 using proprietary algorithms (Xsens, Xsens Technologies BV, Enschede, The Netherlands) and then output and imported into Matlab R2019a (Mathworks Inc., Natick, MA, USA) where the step of interest was extracted, and peak values were calculated for each trial using custom code. Kinematics during the different movements were only investigated if a performance difference was observed during the sprint, agility or vertical jump test (kinematics were not measured during the running testing session). Due to space constraints, kinematics during a single stance phase for each movement were compared—the closest stance phase at the 5 m mark during the sprint, the initial plant leg during the first change in direction during the agility drill and the initial touchdown leg during the jumping movement of the vertical jump and reach test. Peak sagittal plane angles and angular velocities of the ankle, knee, and hip joint, as well as the trunk and center of mass, were compared to the Control condition.

Prior to the experiment participants filled out an initial questionnaire rating their ideal surface properties of cushioning, on a 5-point Likert scale with text anchors of very soft to very hard. Subjective assessments were then collected using a questionnaire that had athletes rank the surfaces on a five-point Likert scale for the following qualities: cushioning (very soft to very hard), cushioning comfort (very uncomfortable to comfortable), perceived athlete performance (very poorly to very well) and overall surface rating (worst surface ever to best surface ever). These assessments were taken during both testing sessions and were recorded immediately following performance on a specific surface.

### 2.4. Statistics

All data was compared to the Control surface using a paired *t*-test at a significance level of α = 0.10 with 95% confidence intervals of the differences (95% CI_Diff_) being reported. Data was formally tested for violation of normality using a Shapiro-Wilk test of normality. The level of significance was chosen as *p* < 0.10 because the consequences of incorrectly accepting a false result (possibly slightly increased expenses for turf owners and manufacturers) are minor in comparison to the benefits of a positive effect (improved athlete performance). The main outcome variables were the specific performance measurements (running economy, sprint time, agility drill time, vertical jump height). If differences in performance occurred, kinematics were investigated and were only compared between surfaces that had significant differences in performance using a paired t-test at a level of α = 0.10. Secondary outcome variables included the subjective assessment measurements on each surface, which were also analyzed using a paired t-test at a level of α = 0.10 [19,20].

Reliability of the timed movements was measured using calculation of the intraclass correlation coefficient and their 95% confidence intervals based on an intra-rater reliability absolute agreement, two-way mixed model. Effect size (Cohen’s d) calculations were made following the method of Cohen (1992) [21] and Dunlap et al. (1996) [22].

All statistical analyses were performed using SPSS software v12.0 (SPSS Inc., Chicago, IL, USA).

## 3. Results

### 3.1. Reliability

Reliability data of the different timed movements is shown in Table 2. All measurements were highly repeatable with no coefficient being below 0.843.

### 3.2. Performance

No data violated the assumption of normality (as evaluated with the Shapiro-Wilks test of normality), performance data are shown in Table 3. The Soft (*p* = 0.069, 90% CI_Diff_ = −0.103 to −1.912) and Softer (*p* = 0.059, 90% CI_Diff_ = −0.154 to −2.021) surfaces resulted in significant reductions in oxygen consumption, both with small effect sizes (0.47 and 0.49, respectively) when compared to the Control surface. No differences were present during the 10 m sprint movement, regardless of surface stiffness. During the agility drill movement, the Softest surface (*p* = 0.031, 90% CI_Diff_ = −0.015 to −0.099) resulted in improved performance compared to the Control surface, with a medium effect size (0.57). Increased vertical jump and reach performance was present when jumping on the Soft surface compared to the Control (*p* = 0.050, 90% CI_Diff_ = 0.265 to 2.666) with a medium effect size (0.60); however, no other surfaces yielded any jumping performance differences.

### 3.3. Athlete Kinematics

During the first cut of the 5-10-5 agility drill, participants had significantly greater center of mass velocity when beginning their change of direction (*p* = 0.069, 90% CI_Diff_ = 0.023 to 0.398), with a medium effect size (0.58), an increased peak ankle plantarflexion angle during the change of direction (*p* = 0.077, 90% CI_Diff_ = −0.192 to −4.60) with a medium effect size (0.54) and a greater trunk lean angle when completing the change of direction (*p* = 0.071, 90% CI_Diff_ = 0.257 to 4.80), with a medium effect size (0.55) on the Softest surface compared to the Control (Table 4).

During the vertical jump, differences in kinematics were observed in both the ankle and knee joint as well as for the center of mass (Table 5). Specifically, the peak ankle dorsiflexion angular velocity (*p* = 0.064, 90% CI_Diff_ = 12.68 to 185.81) with a medium effect size (0.57), and the peak knee extension angular velocity (*p* = 0.074, 90% CI_Diff_ = 2.79 to 58.36) with a medium effect size (0.54) were both increased, as well as the vertical center of mass velocity at takeoff (*p* = 0.038, 90% CI_Diff_ = 0.023 to 0.175), with a medium effect size (0.62) when jumping on the Soft surface compared to the Control.

### 3.4. Subjective Assessments

The subjective assessment of cushioning and cushioning comfort is shown in Table 6. During the sprint, agility and jumping session, no significant differences in subjective ratings were present; however, during the initial running session, participants rated the Stiff surface as being harder than the Control (*p* = 0.083, 90% CI_Diff_ = 0.032 to 1.093) with a medium effect size (0.66) and gave it a lower comfort rating (*p* = 0.088, 90% CI_Diff_ = −0.019 to −0.923) with a medium effect size (0.76).

Participants did not perceive any significant differences in performance between any of the surfaces during the sprinting, agility or jumping movements; however, during the running session participants rated having lower performance on the stiff surface (*p* = 0.070, 90% CI_Diff_ = −0.057 to −1.068) with a large effect size (0.86) (Table 6).

When participants rated the surfaces overall (Table 6), all surfaces were rated equally during the running session, while the Softest surface was rated higher during the sprint/agility/jump session (*p* = 0.068, 90% CI_Diff_ = 0.02 to 0.686) with a medium effect size (0.58).

## 4. Discussion

The main goal of this study was to investigate the influence of surface stiffness of artificial turf on athlete performance. The results of the study indicate that changes in surface stiffness can influence athletic performance, alter kinematics and to a lesser extent subjective assessment of performance, with changes in performance being surface and movement specific.

During steady state running, improvements in running economy of over 2.5% were observed when performing on the Soft surface (16% lower stiffness) and Softer surface (34% lower stiffness). This result supports a previous finding that surface stiffness can influence running performance [5]. During this previous finding, as surface stiffness was decreased (more compliant surfaces) participants altered their leg stiffness and kinematics in order to maintain an overall surface/body stiffness [5,23,24]. Unfortunately, kinematic data was not collected during the steady state running testing session, so insight into the mechanism of these performance increases are limited. However, based on these previous studies, it can be speculated that participants altered their movement patterns on the different surfaces, with the movement patterns on the Soft and Softer surfaces resulting in a decreased metabolic cost.

During the 10 m sprint there were no differences in performance on any of the surfaces. This lack of difference was likely due to the magnitudes of stiffness differences not being substantial enough to elicit performance differences. Data from Gains et al. (2010) [25] would seem to support the idea that larger stiffness differences may be required for sprinting performance differences, as they did not report any performance differences when comparing 40 yard sprint performance on natural grass (stiff) and artificial turf (soft). However, when large changes in stiffness were present (artificial turf vs. stiff indoor lab surface), differences in 5 m sprint time were present [26].

During the cutting agility drill, the softest surface resulted in a significant increase in agility change of direction performance. This supports previous work, which has shown a more compliant surface results in improvements in change of direction performance [25,27]. Examining the kinematics, participants had a greater lean angle while on the softest surface suggesting the increased compliance of the surface was likely to provide a banking effect. Banking has been shown to improve performance [28,29], allowing athletes to lean more and generate larger horizontal forces and center of mass velocity without fear of losing traction. On the Softest surface, athletes entered the cut with greater velocity, were able to utilize the compliance of the Softest surface to alter their velocity and lean further into the next phase of the movement. The improved performance may also have been due to altered traction on the more compliant surface. Unfortunately, outsole traction was not quantified in this study.

Lastly, during the jump and reach movement the soft surface increased performance by 2.4%. When on this surface, participants were able to increase their peak ankle and knee joint angular velocity and as a result their center of mass velocity at takeoff. Apampatzis et al. (2004) [4] reported that, when performing drop jumps on a compliant surface, increased jump performance was due to a higher ratio of positive to negative work. Therefore, when jumping on the Soft surface, the increased joint velocity was likely to lead to an increase in the positive power and mechanical work done by the lower extremity (via an increase in joint power and corresponding work). This speculation would need to be verified. It should be noted that participants were able to perform the jump test with whatever technique that they felt could provide them with the best performance. Due to this freedom, participants either performed a single leg or double legged jumping movement, which may have limited the kinematic analysis, as only a single leg was analyzed. Additionally, the influence of body mass of the participants was not taken into account during the data analysis, with differences in mass of participants potentially leading to differences in surface deformation during the athletic movements.

Subjectively, participants could not discern between any of the reduced stiffness surfaces in terms of surface cushioning; however, the Softest surface was rated closest to their ideal cushioning value (which was softer than the Control). Increasing stiffness of the surface did result in changes in subjective ratings with participants perceiving that the surface was stiffer, rating it as such and also providing this surface with a lower comfort rating. This lower comfort score potentially also resulted in the perception of a decreased performance on this surface [30]. Lastly, during the overall rating of the surfaces, participants rated the Softest surface significantly higher during the short-term performance session (sprint/agility/jump). Overall, these subjective results indicate that during short duration, explosive movements on a surface, participants are not able to significantly differentiate cushioning, comfort or their performance. During longer duration running, participants can only differentiate stiffer surfaces, in terms of cushioning, comfort and performance.

## 5. Conclusions

Overall, in terms of performance, during the running, agility and vertical jump movements, there were specific surfaces that resulted in increased performance. Running was optimized on a Soft and Softer surface; agility was optimized on the Softest surface and jumping was optimized on the Soft surface. Therefore, from the surfaces measured in the current study, if maximizing performance is the desired goal, it is recommended that the Soft surface be used (16% softer than Control, 971 m/s^2^ vs. 1118 m/s^2^). This surface would provide increased performance during running and vertical jumping and would not negatively affect performance during the other movements (sprinting and agility). Based on the data in this study, when selecting surface properties such as stiffness, the main movements on the surface should be taken into consideration; for example, if rapid change of direction movements are commonly performed on the surface, then the Softest surface, with a reduction of 50% from the Control (559 m/s^2^ vs. 1118 m/s^2^), should be selected. Subjectively, participants could not distinguish between any of the softer surfaces in terms of surface cushioning; however, the stiffer surface was rated as harder and less comfortable. Overall, changes in surface stiffness altered athletic performance and, to a lesser extent, subjective assessments of performance, with changes in performance being surface and movement specific.

## Figures and Tables

**Table 1:** Composition of the different surfaces used during testing.

#	Name	Change in Stiffness from Control (%)	Peak Deceleration (m/s^2^)	Carpet Fiber Density	Shock Pad	Bottom Layer	Mix	Top Layer
1	Softest	−50	559	1220 g/m^2^	Expanded Polypropylene (15 mm thick)	4.88 kg/m^2^ sand	25.3 kg/m^2^ sand, 12.7 kg/m^2^ cryo rubber 14–30	1.95 kg/m^2^ cryo rubber 10–14
2	Softer	−34	746	1220 g/m^2^	Cross-linked Polyethylene Foam (24 mm thick)	4.88 kg/m^2^ sand	25.3 kg/m^2^ sand12.7 kg/m^2^ cryo rubber 14–30	1.95 kg/m^2^ cryo rubber 10–14
3	Soft	−16	971	1220 g/m^2^	Therma-plastic (12 mm thick)	4.88 kg/m^2^ sand	25.3 kg/m^2^ sand, 12.7 kg/m^2^ cryo rubber 14–30	1.95 kg/m^2^ cryo rubber 10–14
4	Control	0	1118	1220 g/m^2^	No	4.88 kg/m^2^ sand	25.3 kg/m^2^ sand, 12.7 kg/m^2^ cryo rubber 14–30	1.95 kg/m^2^ cryo rubber 10–14
5	Stiff	+17	1315	915 g/m^2^	No	4.88 kg/m^2^ sand	25.3 kg/m^2^ sand, 12.7 kg/m^2^ cryo rubber 20–50	1.95 kg/m^2^ cryo rubber 14–30

**Table 2:** Intraclass correlation coefficient with 95% confidence intervals in parenthesis for the sprint, agility and jump movements on the five different surfaces.

	Softest	Softer	Soft	Control	Stiff
Sprint	0.867 (0.719–0.948)	0.886 (0.739–0.959)	0.934 (0.853–0.975)	0.886 (0.739–0.959)	0.857 (0.702–0.944)
Agility	0.988 (0.972–0.995)	0.983 (0.960–0.994)	0.868 (0.772–0.949)	0.948 (0.875–0.982)	0.991 (0.981–0.997)
Jump	0.994 (0.983–0.998)	0.991 (0.979–0.997)	0.990 (0.977–0.997)	0.990 (0.973–0.996)	0.988 (0.971–0.996)

**Table 3:** Performance results during the running, sprinting, agility and jump and reach tests. Data represent the mean ± standard deviation of 17 participants, with bold values indicating significant differences from the Control (*p* < 0.10) with ES = Cohen’s d effect size.

	Softest	Softer	Soft	Control	Stiff
Oxygen Consumption (L/min/kg)	38.5 ± 4.6	**38.0 ± 4.8 (ES = 0.49)**	**38.1 ± 4.7 (ES = 0.47)**	39.1 ± 4.3	38.3 ± 5.3
10 m Sprint Time (s)	2.10 ± 0.11	2.10 ± 0.12	2.10 ± 0.13	2.11 ± 0.13	2.11 ± 0.13
5-10-5 Agility Time (s)	**4.95 ± 0.27 (ES = 0.57)**	4.98 ± 0.30	4.95 ± 0.28	5.01 ± 0.27	4.98 ± 0.23
Vertical Jump and Reach Height (cm)	63.0 ± 12.7	63.6 ± 13.9	**63.8 ± 12.8 (ES = 0.60)**	62.3 ± 12.6	63.1 ± 11.7

**Table 4:** Sagittal plane ankle, knee, hip, trunk and center of mass kinematics during the stance phase of the first change of direction of the 5-10-5 agility drill. Data represent the mean ± standard deviation of 17 participants with bold values indicating a significant difference and ES = Cohen’s d effect size.

Joint	Variable	Control	Softest
Ankle	Peak Dorsiflexion Angle (°)	30.9 ± 9.5	31.4 ± 10.3
	Peak Dorsiflexion Angular Velocity (°/s)	927 ± 289	929 ± 281
	Peak Plantarflexion Angle (°)	**24.6 ± 13.3**	**22.2 ± 11.6 (ES = 0.54)**
	Peak Plantarflexion Angular Velocity (°/s)	767 ± 246	794 ± 231
Knee	Peak Flexion Angle (°)	110 ± 7	109 ± 8
	Peak Flexion Angular Velocity (°/s)	962 ± 286	903 ± 216
	Peak Extension Angular Velocity (°/s)	706 ± 292	654 ± 297
Hip	Peak Flexion Angle (°)	75 ± 14	77 ± 20
	Peak Flexion Angular Velocity (°/s)	379 ± 101	346 ± 99
	Peak Extension Angular Velocity (°/s)	605 ± 229	599 ± 224
Trunk	Lean Angle at Takeoff (°)	**3.9 ± 9.8**	**6.4 ± 8.7 (ES = 0.55)**
center of Mass Velocity	center of Mass Velocity at Touchdown (m/s)	**3.3 ± 0.4**	**3.5 ± 0.4 (ES = 0.58)**

**Table 5:** Sagittal plane ankle, knee, hip and center of mass kinematics during the jumping phase vertical jump. Data represent the mean ± standard deviation of 17 participants with bold values indicating a significant difference and ES = Cohen’s d effect size.

Joint	Variable	Control	Soft
Ankle	Peak Dorsiflexion Angle (°)	31.7 ± 7.8	33.2 ± 6.1
	Peak Dorsiflexion Angular Velocity (°/s)	**373 ± 130**	**472 ± 252 (ES = 0.57)**
	Peak Plantarflexion Angle (°)	39.2 ± 7.4	39.9 ± 8.6
	Peak Plantarflexion Angular Velocity (°/s)	1390 ± 242	1444 ± 272
Knee	Peak Flexion Angle (°)	85.9 ± 10.5	87.6 ± 8.1
	Peak Flexion Angular Velocity (°/s)	595 ± 148	601 ± 168
	Peak Extension Angular Velocity (°/s)	**1128 ± 158**	**1159 ± 172 (ES = 0.54)**
Hip	Peak Flexion Angle (°)	63.0 ± 10.7	61.2 ± 10.7
	Peak Flexion Angular Velocity (°/s)	338 ± 100	294 ± 1038
	Peak Extension Angular Velocity (°/s)	548 ± 117	545 ± 129
center of Mass Velocity	center of Mass Velocity at Takeoff (m/s)	**3.2 ± 0.5**	**3.3 ± 0.5 (ES = 0.62)**

**Table 6:** Subjective assessment scores of cushioning, cushioning comfort, perceived performance and overall rating of the different surfaces. Data represent the mean ± standard deviation of 17 participants with bold values indicating a significant difference and ES = Cohen’s d effect size.

	Testing Session	Softest	Softer	Soft	Control	Stiff
Cushioning	Running	2.6 ± 1.1	3.1 ± 0.8	3.5 ± 1.0	3.1 ± 0.9	**3.6 ± 1.0 (Effect Size (ES) = 0.66)**
	Sprint/Agility/Jump	2.6 ± 0.9	2.7 ± 0.8	3.1 ± 0.9	2.8 ± 0.9	2.9 ± 0.9
Cushioning Comfort	Running	3.4 ± 0.8	3.4 ± 1.0	3.4 ± 0.8	3.2 ± 0.7	**2.7 ± 1.2 (ES = 0.76)**
	Sprint/Agility/Jump	3.7 ± 0.9	3.5 ± 0.7	3.3 ± 0.6	3.6 ± 0.6	3.6 ± 0.7
Performance	Running	3.3 ± 1.0	3.1 ± 1.1	3.4 ± 1.1	3.4 ± 1.1	**2.9 ± 1.2 (ES = 0.86)**
	Sprinting	3.6 ± 0.8	3.4 ± 0.9	3.6 ± 0.9	3.5 ± 0.9	3.5 ± 0.8
	Agility	3.2 ± 0.6	2.9 ± 0.8	3.5 ± 0.9	3.1 ± 0.9	3.1 ± 0.8
	Jumping	3.8 ± 0.7	3.4 ± 0.9	3.4 ± 0.8	3.5 ± 0.8	3.7 ± 0.8
Overall Surface Rating	Running	3.3 ± 0.6	3.3 ± 0.9	3.4 ± 0.8	3.3 ± 0.6	2.9 ± 1.0
	Sprint/Agility/Jump	**3.7 ± 0.8 (ES = 0.58)**	3.2 ± 0.9	3.1 ± 0.6	3.4 ± 0.7	3.6 ± 0.8
